# In vitro evaluation of fenfluramine and norfenfluramine as victims of drug interactions

**DOI:** 10.1002/prp2.958

**Published:** 2022-05-22

**Authors:** Parthena Martin, Maciej Czerwiński, Pallavi B. Limaye, Seema Muranjan, Brian W. Ogilvie, Steven Smith, Brooks Boyd

**Affiliations:** ^1^ Zogenix, Inc Emeryville California USA; ^2^ Sekisui XenoTech, LLC Kansas City Kansas USA

**Keywords:** antiepileptics, cytochrome P450, drug transport, drug‐drug interactions

## Abstract

Fenfluramine (FFA) has potent antiseizure activity in severe, pharmacoresistant childhood‐onset developmental and epileptic encephalopathies (e.g., Dravet syndrome). To assess risk of drug interaction affecting pharmacokinetics of FFA and its major metabolite, norfenfluramine (nFFA), we conducted in vitro metabolite characterization, reaction phenotyping, and drug transporter−mediated cellular uptake studies. FFA showed low in vitro clearance in human liver S9 fractions and in intestinal S9 fractions in all three species tested (t_1/2_ > 120 min). Two metabolites (nFFA and an N‐oxide or a hydroxylamine) were detected in human liver microsomes versus six in dog and seven in rat liver microsomes; no metabolite was unique to humans. Selective CYP inhibitor studies showed FFA metabolism partially inhibited by quinidine (CYP2D6, 48%), phencyclidine (CYP2B6, 42%), and furafylline (CYP1A2, 32%) and, to a lesser extent (<15%), by tienilic acid (CYP2C9), esomeprazole (CYP2C19), and troleandomycin (CYP3A4/5). Incubation of nFFA with rCYP1A2, rCYP2B6, rCYP2C19, and rCYP2D6 resulted in 10%−20% metabolism and no clear inhibition of nFFA metabolism by any CYP‐selective inhibitor. Reaction phenotyping showed metabolism of FFA by recombinant human cytochrome P450 (rCYP) enzymes rCYP2B6 (10%–21% disappearance for 1 and 10 µM FFA, respectively), rCYP1A2 (22%−23%), rCYP2C19 (49%−50%), and rCYP2D6 (59%−97%). Neither FFA nor nFFA was a drug transporter substrate. Results show FFA metabolism to nFFA occurs through multiple pathways of elimination. FFA dose adjustments may be needed when administered with strong inhibitors or inducers of multiple enzymes involved in FFA metabolism (e.g., stiripentol).

## Graphical abstract

Reaction phenotyping showed metabolism of the antiseizure medication fenfluramine by recombinant human cytochrome P450 (rCYP) enzymes rCYP2B6, rCYP1A2, rCYP2C19, and rCYP2D6. Fenfluramine dose may need to be adjusted when co‐administered with strong inhibitors or inducers of these enzymes (e.g., stiripentol).

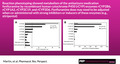

**Abbreviations**
ASM: antiseizure medication
AUC: area under the concentration curve
BCA: bicinchoninic acid
BCRP: breast cancer resistance protein
CDD: CDKL5 deficiency disorder
CID: collision‐induced dissociation
CL_int_
: intrinsic clearance
C_max_
: maximum drug concentration
CYP: cytochrome P450
DDI: drug‐drug interaction
DS: Dravet syndrome
EDTA: ethylenediaminetetraacetic acid
FDA: United States Food and Drug Administration
FFA: fenfluramine
f_m_
: fraction metabolized
HEK: human embryonic kidney cells
k_el_
: rate constant of elimination (min^−^^1^)
LC‐MS/MS: liquid chromatography/tandem mass spectrometry
LGS: Lennox‐Gastaut syndrome
m/z: mass‐to‐charge ratio
MDCKII: Madin‐Darby canine kidney cells
MS/MS: tandem mass spectrometry
NADP: nicotinamide adenine dinucleotide phosphate
NADPH: nicotinamide adenine dinucleotide phosphate (hydrogen)
nFFA: norfenfluramine
OAT: organic anion transporter
OCT: organic cation transporter
P‐gp: P‐glycoprotein multidrug transporter
rCYP: recombinant cytochrome P450
t_1/2_
: half‐life (in vitro)
TEER: transepithelial electrical resistance
t_R_
: retention time
UDPGA: uridine diphosphate glucuronic acid
UGT: uridine diphosphate glucuronosyltransferase

**Significance statement**

Fenfluramine reduced convulsive seizure frequency in patients with Dravet syndrome and other developmental and epileptic encephalopathies. These patients take multiple concurrent antiseizure medications, emphasizing the importance of evaluating fenfluramine’s drug‐drug interaction potential. We characterized the victim potential of fenfluramine and its active metabolite, norfenfluramine, by reaction phenotyping. Fenfluramine dose adjustments may be needed when administered with strong inhibitors or inducers of CYP2D6, CYP2B6, CYP1A2 and, to a lesser extent, CYP2C9, CYP2C19, and CYP3A4/5. A companion paper evaluates fenfluramine’s perpetrator potential.

## 1 INTRODUCTION

Fenfluramine (FFA), as designated by International Union of Pharmacology (IUPHAR),^1^ or (RS)‐ethyl (α‐methyl‐3‐trifluoromethylphenethyl)amine, and its major metabolite, norfenfluramine (nFFA), modulate serotonergic neurotransmission. In clinical trials, FFA has shown efficacy for treatment of seizures associated with Dravet syndrome (DS), Lennox‐Gastaut syndrome (LGS), and CDKL5 deficiency disorder (CDD). All are pediatric developmental and epileptic encephalopathies characterized by pharmacoresistant seizures of multiple types.^2^, ^3^, ^4^, ^5^ FFA has been approved in the US, EU, and UK for the treatment of DS, and was recently approved for Lennox‐Gastaut syndrome in the US (Prescribing information: https://www.fintepla.com/). The severity and pharmacoresistance of these conditions often require multi−ASM regimens. Resultant drug‐drug interactions (DDIs) may lead to loss of efficacy or to toxicity by reducing or elevating plasma drug levels to subtherapeutic or toxic concentrations, respectively.^6^

Characterizing the metabolic stability, metabolites, and substrate potential for drug transporters of antiseizure medications (ASM) and the enzymes that catalyze their biotransformation and elimination can help predict pharmacokinetic DDIs. Early literature dating to the 1960s and 1970s—when FFA was marketed as an anorectic agent—describes FFA and nFFA metabolism in multiple species,^7^, ^8^, ^9^, ^10^, ^11^, ^12^ but the clinical pharmacology of FFA and nFFA has not been systematically described using updated protocols to predict potential clinical DDIs according to EMA and FDA guidelines.^13^, ^14^

Seven cytochrome P450 (CYP450) enzymes mediate phase 1 biotransformation and elimination reactions for most orally administered xenobiotics in the liver and, to a lesser extent, in the intestine.^15^ Reaction phenotyping studies in liver microsomes can reveal the key CYP450 enzymes catalyzing metabolic transformation, allowing prediction of pharmacokinetic DDIs.

In the context of ASM polypharmacy, ASMs with fraction metabolized (f_m_) >25% by a single enzyme or clearance pathway have high victim potential.^14^, ^16^ ASMs that are substrates of drug transporters are potential DDI victims. Earlier reports document FFA and nFFA metabolism,^9^, ^12^, ^17^, ^18^ but FFA metabolism has not been fully characterized in the context of predicting DDIs when used as an add‐on to existing ASM regimens in patients with DS or LGS. In vitro studies conducted according to DDI guidance have not yet been reported.^13^, ^14^ Developed by experts in drug metabolism, the FDA and EMA guidance documents were compiled based on the most current state of the science for predicting DDI. The guidelines constitute “a systematic, risk‐based approach to assessing DDI potential of investigational drugs and making recommendations to mitigate DDIs.”^14^ Polypharmacy is common in treating developmental and epileptic encephalopathies; FFA is likely to be used in combination ASM regimens. The potential for DDIs must be fully evaluated, and the rigorous, systematic guidelines provided by the FDA and EMA represent their current thinking on the studies necessary to predict DDIs in combination ASM regimens.

To predict the victim potential of FFA and nFFA, we performed metabolic stability, metabolite identification, reaction phenotyping, and drug transporter substrate studies in vitro. Metabolites formed were identified in rat, dog, and human liver and intestinal S9 fractions by liquid chromatography/tandem mass spectrometry (LC‐MS/MS). CYP enzymes mediating FFA and nFFA metabolism were identified via recombinant proteins and direct or metabolism‐dependent inhibitors. A companion manuscript characterizes the perpetrator potential of FFA.^19^

## 2 MATERIALS AND METHODS

### 2.1 Chemicals

Diethyldithiocarbamate trihydrate, ethylenediaminetetraacetic acid (EDTA), furafylline, glucose‐6‐phosphate, glucose‐6‐phosphate dehydrogenase, ketoconazole, letrozole, magnesium chloride, nicotinamide adenine dinucleotide phosphate (NADP), paroxetine maleate salt, phencyclidine hydrochloride, quinidine, sucrose, and tris(hydroxymethyl)aminomethane hydrochloride, or tromethane hydrochloride base, were obtained from Sigma‐Aldrich (chemicals company), in St. Louis, Missouri. The deuterium‐labeled metabolites used as an internal standard for nFFA, hydroxybupropion‐d_6_, gemfibrozil glucuronide, and esomeprazole were received from Toronto Research Chemicals, Inc. (Toronto, Ontario, Canada). Tienilic acid was purchased from Cypex, Ltd., in Dundee, Scotland, and troleandomycin triacetate from Enzo Life Sciences, Inc., in Farmingdale, New York. The deuterium‐labeled metabolite used as the internal standard for FFA was 1’‐hydroxymidazolam‐d_4_ (Cerilliant Corporation, Round Rock, TX). All other reagents and solvents were of analytical grade. FFA and nFFA, or 1‐(3‐(trifluoromethyl)phenyl)propan‐2‐amine hydrochloride, were obtained from IPCA Onyx Scientific Ltd. (Sunderland, UK).

### 2.2 Biological test materials

Microsomes from a mixed‐gender pool of 200 non‐transplantable human livers (Sekisui XenoTech, LLC, Kansas City, KS) were characterized as described.^20^, ^21^ Human recombinant CYP450s (rCYP450s) and control membranes from *Escherichia coli* transfected with empty expression plasmid or plasmid expressing nicotinamide adenine dinucleotide phosphate (hydrogen) (NADPH)‐CYP450 oxidoreductase without CYP450 enzymes were obtained from Cypex, Ltd. Metabolite identification and stability studies used human liver S9 fractions (containing both microsomal and cytosolic fractions of the cell) from pooled human liver (*n* = 50) and intestine (*n* = 10), pooled canine liver (*n* = 8) and intestine (*n* = 7), and pooled rat liver (*n* = 454) and intestine (*n* = 199). All rCYP450 enzymes except rCYP1A2 and rCYP2D6 were expressed with cytochrome b5.

### 2.3 LC‐MS/MS analytical methods

LC‐MS/MS analyses to assess metabolic stability and identify the transporter substrate were performed using an API 4000 QTRAP mass spectrometer (SCIEX, Framingham, MA) with electrospray ionization in positive mode. Chromatography was performed using an Atlantis dC18 analytical column (100 × 2.1 mm, 5 µm; Waters Corporation, Milford, MA). Mobile phases were 0.2% v/v formic acid in water and 0.2% v/v formic acid in acetonitrile. For metabolic stability experiments, FFA and metabolites were eluted at a flow rate of 0.6 mL/min, with a linear gradient of 20%−80% acetonitrile from 0.30−2.0 min. For transporter substrate experiments, the gradient was slightly modified to achieve suitable chromatography. The mass transitions monitored were 232.0 > 159.0 for FFA and 348.0 > 330.0 for 1ʹ‐OH‐midazolam‐d_4_ internal standard.

For metabolic characterization experiments, LC‐MS/MS analyses were performed using an Acquity Ultra Performance Liquid Chromatography system with an in‐line photodiode array detector and ethylene bridged hybrid C18 columns (2.1 × 100 mm, 1.7 µm; Waters Corporation) coupled to a Synapt G2 HDMS QTOF (Waters Corporation). Mobile phases were 0.1% v/v formic acid in water and 0.1% v/v formic acid in methanol. FFA and metabolites were eluted at a flow rate of 0.5 mL/min with a linear gradient of 2.0%−35% methanol from 1.0−10.0 min. The mass spectrometer was operated in positive mode with electrospray ionization. Data were acquired over the range 50−1200 m/z (mass‐to‐charge ratio) using capillary voltage, 3.5 kV; extraction cone voltage, 4.0 V; source temperature, 120°C; and desolvation temperature, 350°C. A continuous lock mass reference compound, fexofenadine, was sampled at 10‐s intervals for centroid data mass correction. Data were processed with MetaboLynx XS, a component of MassLynx (v. 4.1) software (Waters Corporation).

For FFA and nFFA reaction phenotyping experiments, LC‐MS/MS experiments were performed using an API 4000 QTRAP or QTRAP 5500 mass spectrometer (SCIEX) with positive electrospray ionization. Chromatography was performed using an Atlantis dC18 analytical column (100 × 2.1 mm, 5 µm; Waters Corporation). Mobile phases were 0.2% v/v formic acid in water and 0.2% v/v formic acid in methanol. FFA and nFFA were eluted at a flow rate of 0.5 mL/min with a linear gradient of 40%−95% methanol from 0.20−2.1 min. The mass transitions monitored were 232.0>109.0 for FFA, 204.0>159.0 for nFFA, 348.0>330.0 for 1’‐hydroxymidazolam‐d4 internal standard (FFA reaction phenotyping), and 262.0>244.0 for hydroxybupropion‐d6 internal standard (nFFA reaction phenotyping). Analyst Instrument Control and Data Processing software (v. 1.6.1) was used for collecting and integrating data (SCIEX). Calibration curves ranging from 1.25 µM−0.01 µM were used to quantify nFFA. Relative quantitation was used to approximate the amount of FFA test material used.

### 2.4 LC‐MS/MS data analysis and processing

The line of best‐fit for calibration standards was calculated by weighted (1/x) linear regression based on peak‐area ratios of analyte to the internal standard using Analyst Instrument Control and Data Processing software (version 1.6.1; SCIEX). Mean analyte peak‐area ratio at each time point was normalized to controls at t = 0 min (100%).

### 2.5 Metabolic stability and metabolite identity

FFA (1 or 10 µM) was incubated with liver and intestinal S9 fractions (2 mg protein/mL) at 37 ± 1°C in 0.2‐mL incubation mixtures containing potassium phosphate buffer (50 mM, pH 7.4), magnesium chloride (MgCl_2_) (3 mM), and EDTA (1 mM, pH 7.4), in the presence or absence of a three‐component cofactor mixture (NADPH‐generating system, uridine diphosphate glucuronic acid (UDPGA) [8 mM], and 3’‐phosphoadenosine 5’‐phosphosulfate [0.5 mM]). The NADPH‐generating system consisted of NADP (1 mM, pH 7.4), glucose‐6‐phosphate (5 mM, pH 7.4), and glucose‐6‐phosphate dehydrogenase (1 U/mL). Reactions were initiated by adding the cofactor mix and were stopped at 0, 30, 60, or 120 min by adding an equal volume of acetonitrile. Cofactor controls were incubations performed in parallel in the absence of cofactor for 120 min. Additional incubations were performed with midazolam (10 μM) as a positive control to establish the metabolic competence of test systems. Samples were centrifuged at 920 × g for 10 min at 10°C. Supernatant fractions were analyzed by LC‐MS/MS.

To characterize substrate loss and metabolic stability, duplicate samples were analyzed by LC‐MS/MS to determine the change in substrate concentration over the incubation period. The rate constant of elimination (k_el_, min^−1^) was determined from the time course of substrate loss based on single exponential decay ${\text{(A}}_{\left(t\right)}=A_{0}^{\left(‐k_{\text{el}}^{t}\right)})$. Half‐life in vitro was determined by the following equation: t_1/2_ = 0.693/k_el_. The in vitro intrinsic clearance (CL_int_) was calculated from the rate constant of elimination as follows:

$$\begin{array}{c} {\text{CL}}_{\text{int}}\left(\mu L/\text{min}/\text{mg}\;\text{protein}\right)=k_{\text{el}}\left({\text{min}}^{‐1}\right)\times \frac{\text{incubation}\;\text{volume}\;(\mu L)}{\text{mg}\;\text{protein}\;\text{per}\;\text{incubation}}. \end{array}$$

Percent loss of substrate was determined by normalizing the mean analyte peak‐area ratio at each time point to the average of four replicates at the 0‐min time point (100%).

### 2.6 Reaction phenotyping: rCYP450s

Methods used to evaluate the role of CYP450s in the metabolism of FFA have been described.^22^, ^23^, ^24^ To determine whether FFA and nFFA were substrates for specific CYP450s, either FFA (1 and 10 μM) or nFFA (0.1 and 1 μM) was incubated via a Tecan Liquid Handling System (Tecan Life Sciences, Männedorf, Switzerland) in duplicate with 25 pmol of rCYP1A2, rCYP2B6, rCYP2C8, rCYP2C9, rCYP2C19, rCYP2D6, or rCYP3A4, and incubation at 37°C. The 200‐μL incubation mixtures contained potassium phosphate buffer (50 mM, pH 7.4), MgCl_2_ (3 mM), and EDTA (1 mM, pH 7.4), and an NADPH‐generating system. Incubations of FFA with control bactosomes and membranes containing human NADPH‐CYP450 oxidoreductase, but not human CYP450 (reductase control), served as negative controls. FFA or nFFA was added to the incubation mixtures in water (1% v/v). Reactions were initiated by adding the NADPH‐generating system and were terminated at 0 and 60 min by adding 175 μL acetonitrile stop reagent containing an internal standard (1’‐hydroxymidazolam‐d_4_ [final concentration of 50 ng/mL] for FFA; hydroxybupropion‐d_6_ [final concentration of 10 ng/mL] for nFFA). At termination, each sample volume was normalized to the volume of standards (400 μL) with 25 μL of standard blank (methanol). Samples were centrifuged (920 × g for 10 min at 10°C). Supernatant fractions were analyzed by LC‐MS/MS.

### 2.7 Chemical inhibition of FFA and nFFA metabolism

To determine the metabolic role of individual CYP450 enzymes more quantitatively, FFA (1 μM) or nFFA (0.1 μM) was manually incubated in duplicate with human liver microsomes (1 mg protein/mL) for 60 min (FFA), or for 0 and 120 min (nFFA), in the presence of direct‐acting or metabolism‐dependent chemical inhibitors. Direct‐acting chemical inhibitors were letrozole (CYP2A6; 10 µM in 0.5% v/v acetonitrile), quinidine (CYP2D6; 0.1, 1, and 10 µM in water), and ketoconazole (CYP3A4/5; 0.1, 0.5, and 1 µM in 0.5% v/v acetonitrile). Metabolism‐dependent inhibitors were furafylline (CYP1A2; 10 µM in 0.5% v/v acetonitrile), phencyclidine (CYP2B6; 30 µM in water), gemfibrozil glucuronide (CYP2C8; 100 µM in 1% v/v acetonitrile with 0.1% formic acid), tienilic acid (CYP2C9; 20 µM in 0.5% v/v acetonitrile), esomeprazole (CYP2C19; 10 µM in 0.5% v/v 40:60 methanol:Tris pH 9), paroxetine (CYP2D6; 5 µM in water), diethyldithiocarbamate (CYP2E1; 10 µM in water), and troleandomycin (CYP3A4/5; 50 µM in 0.5% v/v acetonitrile). Solvent controls were processed in parallel with each inhibitor. Under the conditions described above, incubations were conducted and reactions terminated at 0 or 120 min. Metabolite formation for chemical inhibitors was normalized over time to metabolite formation in the respective solvent controls for each inhibitor. Assays were conducted in duplicate due to the low biological and technical variability expected in this assay; statistical analysis was not possible with duplicate samples.

### 2.8 Efflux and uptake assays in transfected cells for transporter substrate determination

FFA and nFFA were evaluated as substrates of the transporters breast cancer resistance protein (BCRP), organic anion transporter (OAT)1, OAT3, organic cation transporter (OCT)2, MATE1, MATE2‐K, and P‐glycoprotein multidrug transporter (P‐gp; also known as MDR1 and ABCB1), according to published methods.^25^, ^26^, ^27^, ^28^, ^29^ Madin‐Darby canine kidney (MDCKII)‐BCRP cells (Netherlands Cancer Institute, Amsterdam, Netherlands) or human embryonic kidney (HEK)293 cells transfected with other transporters (Sekisui Medical Co., Ltd., Tokyo, Japan) were used. Transporter‐transfected and control cells were incubated with FFA or nFFA (0.1, 1, or 10 μM), and the amount of FFA and nFFA accumulated in the cells was measured by LC‐MS/MS as described above. The accumulation of positive control substrates in the presence and absence of an inhibitor served as a positive control for transporter function. Control probes and inhibitors for drug transporter−transfected cell lines were as follows: for MDCKII‐BCRP, prazosin substrate control probe (1 µM) with Ko143 (1 µM) inhibitor; for HEK293‐OAT1, [^3^H]‐p‐aminohippurate probe (1 μM) and probenecid inhibitor (100 μM); for HEK293‐OAT3, [^3^H]‐estrone‐3‐sulfate probe (50 nM) and probenecid inhibitor (100 μM); for HEK293‐OCT2, [^14^C]‐metformin probe (10 μM) and quinidine inhibitor (300 µM); and for HEK293‐MATE1 or HEK293‐MATE2‐K, [^14^C]‐metformin probe (10 μM) and cimetidine inhibitor (MATE1, 10 μM; MATE2‐K, 100 μM) or pyrimethamine inhibitor (0.3 μM). BCRP substrate determination was made by bi‐directional permeability across MDCKII‐BCRP and control cells cultured on 24‐well transwell plates. Incubation medium containing probe substrate was added. Ten minutes later, transepithelial electrical resistance (TEER) was recorded and cells were preincubated at 37 ± 2°C for 30−60 min before FFA or nFFA or positive control substrate with the solvent control or positive control inhibitor was added to the donor (apical) chamber. Incubation medium with solvent control or positive control inhibitor was added to the receiver (basolateral) chamber, and aliquots were collected from the donor (20 µL) at 0 and 120 min, or from receiver compartments (100 µL) at 0, 15, 30, and 120 min, and were replaced with comparable volumes of incubation medium. Samples containing FFA, nFFA, or probe substrate were mixed with internal standard and analyzed by LC‐MS/MS. For OAT‐, OCT‐, and MATE‐HEK293 cells, substrate was identified by determining the accumulation of FFA and nFFA in transporter‐expressing cells. After incubation with FFA, nFFA, or radiolabeled positive control substrate in the presence or absence of positive control inhibitor, incubation medium was removed and cells were rinsed with 1 mL of ice‐cold phosphate buffered saline (0.2% bovine serum albumin), washed, and for radiolabeled compounds, resuspended in sodium hydroxide (0.1 M). An aliquot was added to 96‐well plate containing scintillation fluid that was analyzed by a MicroBeta^2^ scintillation counter (Perkin Elmer, Waltham, MA). Protein concentration was determined by a bicinchoninic acid (BCA) protein assay according to the manufacturer’s protocol (Thermo Fisher Scientific, Waltham, MA). Non‐radiolabeled compounds were resuspended in a 50:50 v/v methanol:water solution containing internal standard and were analyzed by LC‐MS/MS. Assays were run in triplicate.

### 2.9 Nomenclature of targets and ligands

Key protein targets and ligands in this article are hyperlinked to corresponding entries in http://www.guidetopharmacology.org, the common portal for data from the IUPHAR/BPS Guide to PHARMACOLOGY,^30^ and are permanently archived in the Concise Guide to PHARMACOLOGY 2021/22^31^, ^32^, ^33^ and the IUPHAR/BPS Guide to Pharmacology Database.^1^

## 3 RESULTS

### 3.1 Metabolic stability

**FIGURE 1:**
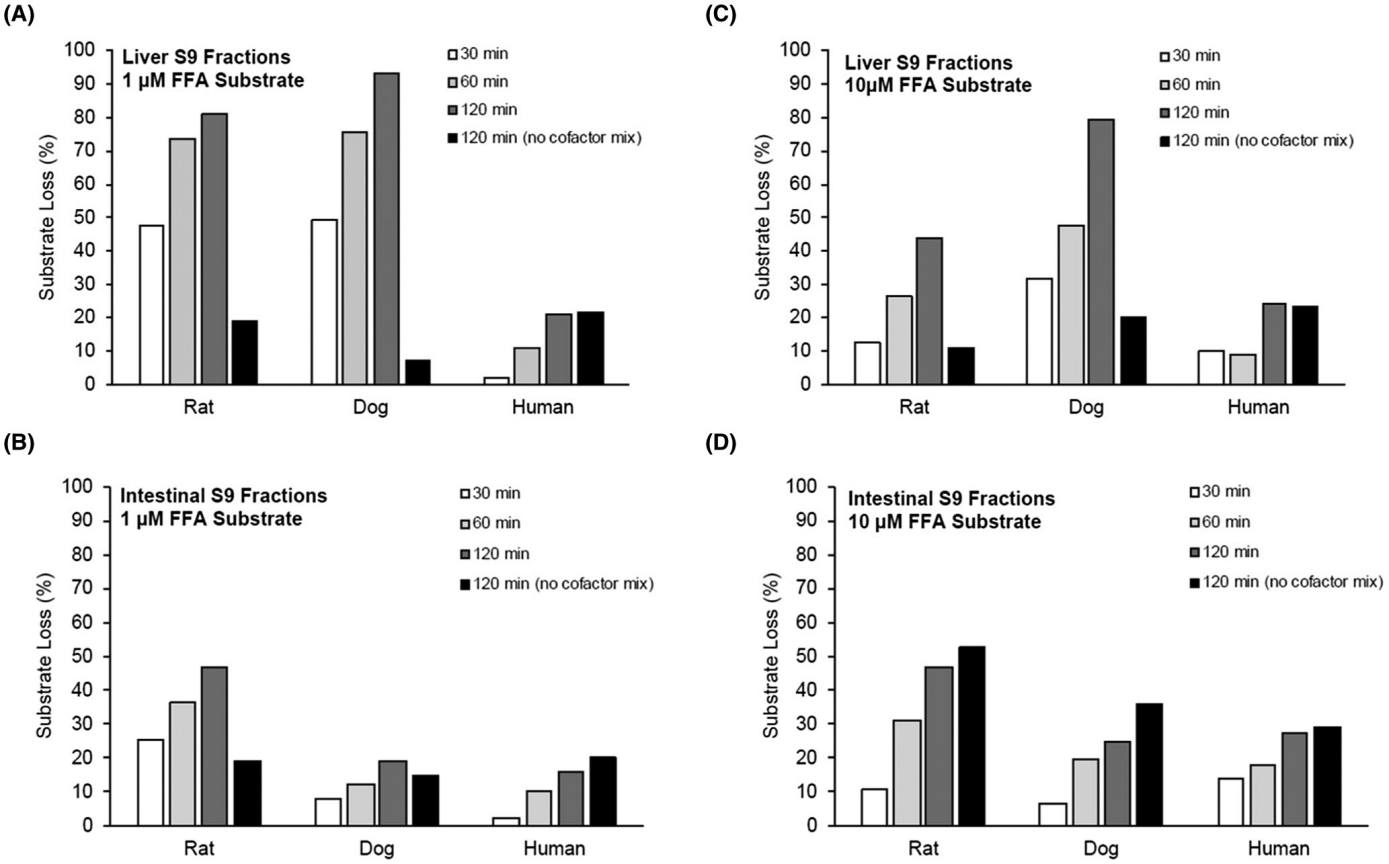
Metabolic stability (percentage substrate loss) of 1 µM Fenfluramine (FFA) in (A) liver S9 fractions or (B) intestinal S9 fractions from rat, dog, or human over time in the presence or absence of NADPH‐containing cofactor mix. Loss of 10 µM FFA substrate in liver (C) and intestinal (D) S9 fractions from rat, dog, and human in the presence or absence of NADPH/cofactors. FFA, fenfluramine; NADPH, nicotinamide adenine dinucleotide phosphate (hydrogen)

FFA was metabolized by rat and dog liver S9 fractions but showed low in vitro intrinsic clearance in human liver S9 fractions and intestinal S9 fractions of all species (Figure 1). At a concentration of 1 µM FFA, rat, dog, and human liver S9 fractions (2 mg protein/mL) showed a time‐dependent FFA substrate loss in all species (Figure 1A). Rates of 1 μM FFA substrate loss followed this rank order in liver S9 fractions: rat (t_1/2_ = 35.4 min; CL_int_ = 9.8 µL/min/mg) ≈ dog (t_1/2_ = 30.0 min; CL_int_ = 11.6 µL/min/mg) >> human (outside assay detection limits t_1/2_ > 120 min; CL_int_ < 2.9 µL/min/mg). Substrate loss in rat and dog but not in human S9 liver fractions was NADPH‐dependent (Figure 1A). Although t_1/2_ and CL_int_ for intestine S9 fractions were outside assay detection limits for all species after 1 µM FFA (t_1/2_ > 120 min; CL_int_ < 2.9 µL/min/mg), rat intestinal S9 fractions showed slightly more substrate loss than other species (Figure 1B). Rat and dog liver S9 fractions showed NADPH‐dependent substrate loss of FFA (Figure 1A). The overall percent of FFA substrate loss in liver S9 fractions after 10 µM FFA was approximately half of substrate loss after 1 µM FFA for all species (compare Figure 1A and 1C). At 10 μM, substrate loss was also dependent on NADPH in rat and dog but not human liver S9 fractions (Figure 1C). In both rat and dog liver S9 fractions at 10 µM FFA, there was approximately twice as much NADPH‐independent FFA substrate loss compared to NADPH‐dependent substrate loss (Figure 1C). In human S9 liver fractions, both overall percentage of substrate loss and proportion of NADPH‐independent substrate loss were comparable at 1 and 10 µM FFA (Figure 1A, 1C). In dog intestinal S9 fractions, percentage of FFA substrate loss was about 2‐fold higher at 10 µM than at 1 µM; no species showed NADPH‐dependent FFA substrate loss at 10 µM in intestinal S9 fractions (Figure 1D). Overall, FFA demonstrated more NADPH‐independent metabolism in human liver and S9 intestinal fractions compared with rat and dog. In vitro CL_int_ was lower in human liver S9 fractions than rat or dog S9 fractions, and FFA clearance was uniformly low in rat, dog, and human intestinal S9 fractions.

### 3.2 Metabolite identity

**FIGURE 2:**
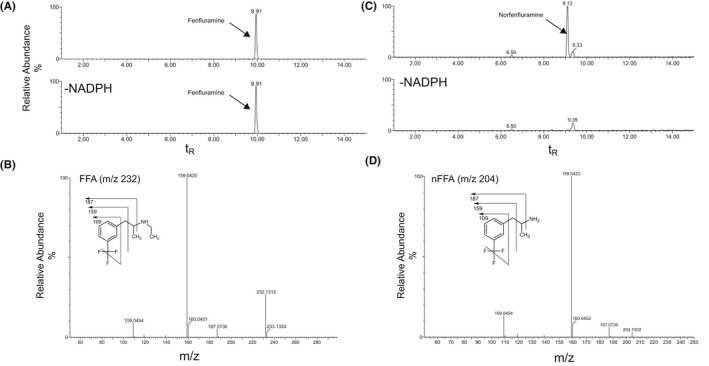
Identification of Fenfluramine (FFA) and norfenfluramine (nFFA) in 120‐min incubations of human liver S9 fraction (representative spectra). (A) Extracted ion chromatogram of FFA in the presence or absence of NADPH‐generating system (120 min). (B) CID MS/MS spectrum of 2 µM FFA reference standard (m/z 232; t_R_ = 9.9 min). (C) Representative extracted ion chromatogram of nFFA in the presence or absence of NADPH‐generating system. (D) CID MS/MS spectrum of 2 µM nFFA reference standard (m/z 204; t_R_ = 9.12 min). CID, collision‐induced dissociation; FFA, fenfluramine; MS/MS, tandem mass spectrometry; m/z, mass‐to‐charge ratio; NADPH, nicotinamide adenine dinucleotide phosphate (hydrogen); nFFA, norfenfluramine; t_R_, retention time

FFA, its de‐alkylated metabolite, nFFA, and six other FFA‐related metabolites (C1−C6) were detected (Table 1). FFA and nFFA were present in extracted ion chromatograms from rat, dog, and human liver and in intestinal S9 fractions at retention times of 9.91 and 9.12 min, respectively (Table 1; Figure 2). FFA was present in incubations with and without NADPH (Figure 2A), but the appearance of the nFFA peak required NADPH in all species and in both liver and intestinal S9 fractions (Figure 2C). Further fragmentation of these parent compounds resulted in product ions of m/z 187, 159, and 109; an additional peak at the parent mass of m/z 232 was present in the FFA but not the nFFA spectra (Figure 2B, D).

**TABLE 1:** FFA metabolites identified in rat, dog, or human liver or intestinal S9 fractions

Parent metabolite	t_R_ (min)	Parent, Metabolite (m/z)	Proposed biotransformation	Liver S9 fractions			Intestinal S9 fractions		
				Rat	Dog	Human	Rat	Dog	Human
FFA	9.93	232	–	X	X	X	X	X	X
nFFA	9.13	204	N‐Dealkylation	X	X	X	X	X	X
C1	3.95	424	Hydroxylation + glucuronide conjugation	X					
C2	10.77	220	N‐Dealkylation + N‐oxygenation to yield an N‐oxide, or N‐hydroxylation to yield a hydroxylamine	X	X	X	X		
C3	11.65	394	N‐Dealkylation + oxygenation + dehydrogenation + glucuronide conjugation	X	X				
C4	12.48	218	N‐Dealkylation + oxygenation + dehydrogenation	X	X				
C5	12.55	218	N‐Dealkylation + oxygenation + dehydrogenation	X	X				
C6	12.71	424	Oxygenation + glucuronide conjugation	X	X				

Abbreviation: FFA, fenfluramine; m/z, mass‐to‐charge ratio; t_R_, retention time.

**FIGURE 3:**
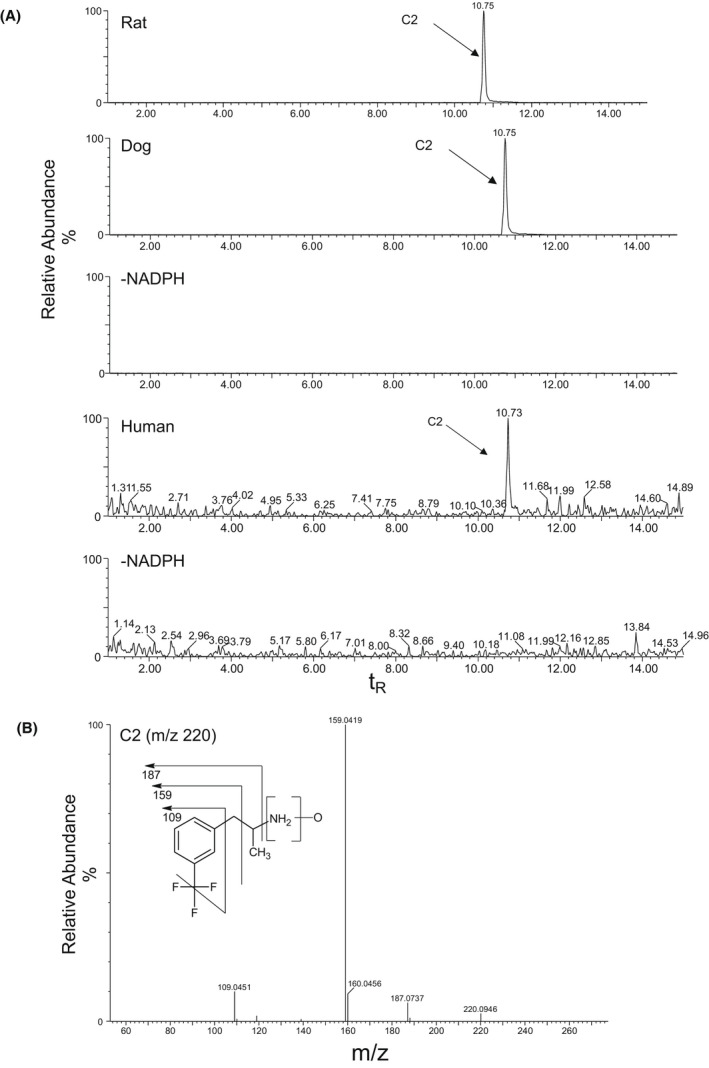
Identification of C2 in 120‐min incubations of Fenfluramine (FFA) (10 µM) with rat, dog, or human liver S9 fractions (2 mg protein/mL). (A) Extracted ion chromatogram of C2 from rat, dog, or human liver S9 fractions in the presence or absence of NADPH‐generating system. (B) CID MS/MS spectrum of C2 (m/z 220; t_R_ = 10.8 min). CID, collision‐induced dissociation; FFA, fenfluramine; MS/MS, tandem mass spectrometry; m/z, mass‐to‐charge ratio; NADPH, nicotinamide adenine dinucleotide phosphate (hydrogen); t_R_, retention time. In the absence of NADPH, no C2 peak was observed in any species.

**FIGURE 4:**
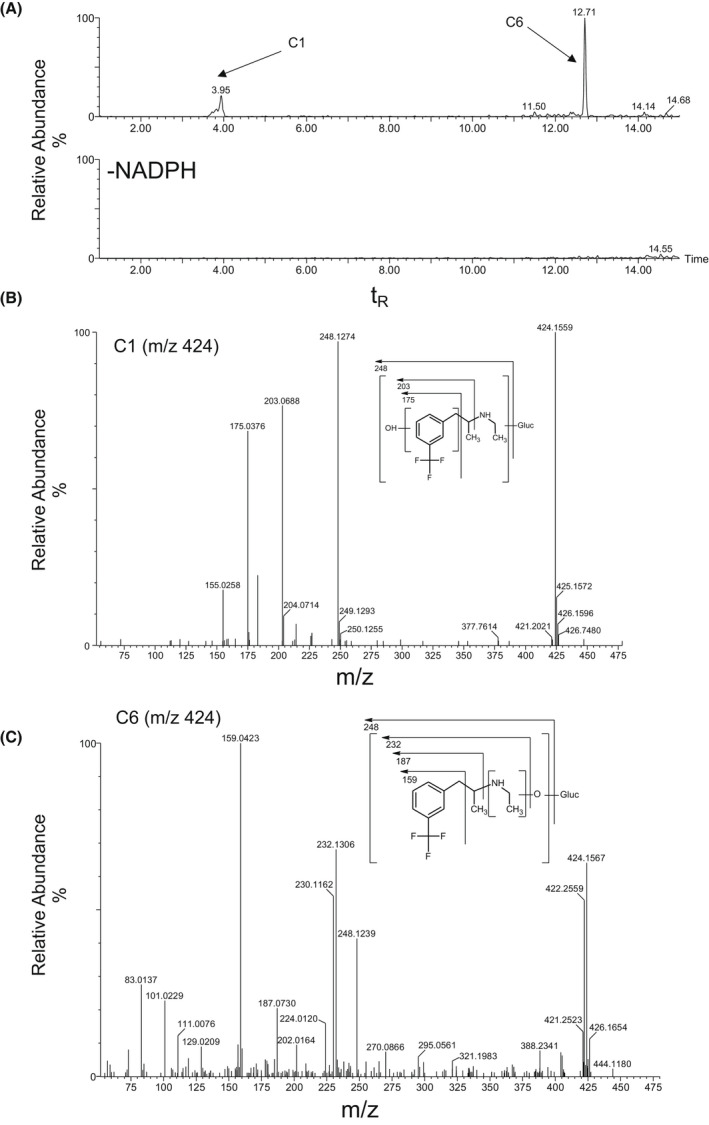
Identification of C1 and C6 in 120‐min incubations of Fenfluramine (FFA) (10 µM) with rat liver S9 fraction (2 mg protein/mL). (A) Extracted ion chromatogram of FFA metabolites formed in the presence or absence of NADPH‐generating system. (B) CID MS/MS spectrum of FFA (m/z 424; t_R_ = 4.0 min) in C1. (C) CID MS/MS spectrum of FFA (m/z 424; t_R_ = 12.7 min) in C6. CID, collision‐induced dissociation; FFA, fenfluramine; MS/MS, tandem mass spectrometry; m/z, mass‐to‐charge ratio; NADPH, nicotinamide adenine dinucleotide phosphate (hydrogen); t_R_, retention time

**FIGURE 5:**
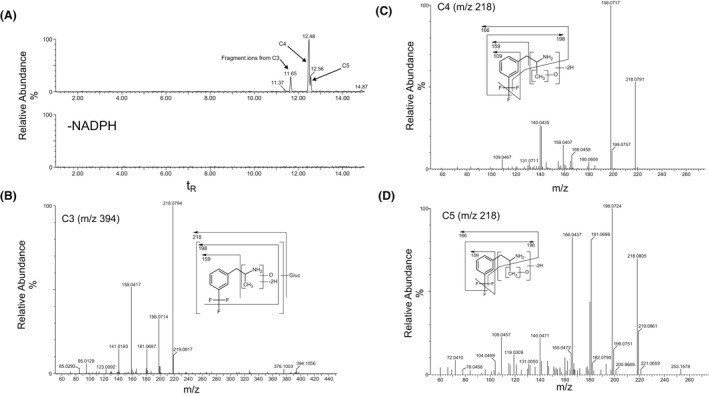
Identification of C3, C4, and C5 in 120‐min incubations of Fenfluramine (FFA) (10 µM) with rat liver S9 fraction (2 mg protein/mL). (A) Extracted ion chromatogram of FFA metabolites formed in the presence or absence of NADPH‐generating system. (B) CID MS/MS spectrum of C3 (m/z 394; t_R_ = 11.7 min). (C) CID MS/MS spectrum of C4 (m/z 218; t_R_ = 12.5 min). (D) CID MS/MS spectrum of C5 (m/z 218; t_R_ = 12.6 min). CID MS/MS spectrum of norfenfluramine (nFFA) (m/z 204; t_R_ = 9.1 min). CID, collision‐induced dissociation; FFA, fenfluramine; MS/MS, tandem mass spectrometry; m/z, mass‐to‐charge ratio; NADPH, nicotinamide adenine dinucleotide phosphate (hydrogen); nFFA, norfenfluramine; t_R_, retention time

Overall, the metabolites of FFA were formed by N‐dealkylation, oxygenation, and dehydrogenation, or a combination thereof, with and without glucuronide conjugation. nFFA formed by N‐dealkylation (N‐de‐ethylation) of FFA was observed in all liver and intestinal fractions (Table 1). FFA and nFFA were observed after 120‐min incubations of human liver S9 fractions (Figure 2). nFFA was further metabolized to form components C2 through C5 in both dog and rat liver S9 fractions, C2 in human liver S9 fractions, and trace C2 in rat intestinal S9 fractions. C2 was formed from FFA by N‐dealkylation followed by N‐oxygenation or N‐hydroxylation to yield either an N‐oxide or a hydroxylamine in liver fractions from all species (Figure 3); C1 was formed from FFA by hydroxylation and glucuronide conjugation in rat liver S9 fractions only (Figure 4A, B). C6 was detected in the same fractions as C1 in the rat and was formed by oxygenation and glucuronide conjugation (Figure 4A, C). C3 was formed by a combination of N‐dealkylation, oxygenation, dehydrogenation, and glucuronide conjugation (Figure 5A, B). Fragment ions for C3 appeared in the same fractions as C4 and C5, which were formed by a combination of N‐dealkylation, oxygenation, and dehydrogenation (Figure 5A, C, D). All metabolites required NADPH‐dependent transformation (120 min) before conjugation (Figures 3–5). nFFA and its subsequent N‐oxygenation product (C2) were the only FFA metabolites detected in human liver microsomes (Table 1), and nFFA was the only metabolite detected in human intestinal microsomes (Table 1). The FFA metabolites detected in human liver and intestine were also observed in both rat and dog. Evidence of hydroxylation, dehydrogenation, and glucuronidation was observed in rat and dog but not human test systems. Glucuronide conjugation was observed in rat and dog but not human liver S9 fractions in the fragmentation patterns of C3 and C6 (Figure 4, Figure 5). No human‐specific metabolites were detected (Table 1).

### 3.3 FFA reaction phenotyping

**FIGURE 6:**
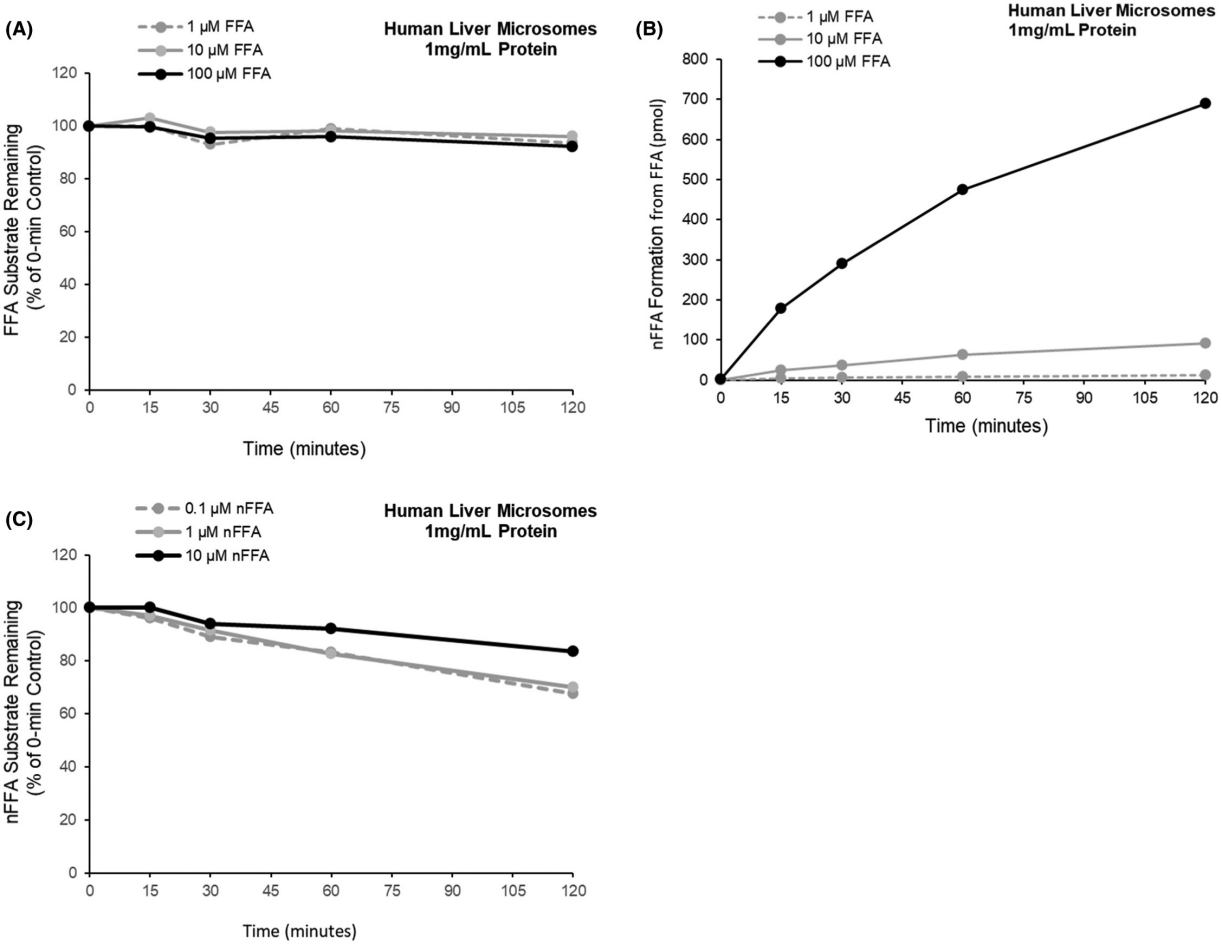
Fenfluramine (FFA) substrate loss, norfenfluramine (nFFA) formation, and nFFA substrate loss in human liver microsomes (1 mg/mL protein). (A) NADPH‐dependent FFA loss in incubations of 1, 10, or 100 µM substrate. (B) NAPDH‐dependent nFFA formation in incubations of 1, 10, or 100 µM FFA. (C) nFFA loss in incubations of 0.1, 1, or 10 µM substrate. Points represent the mean of n=2 replicates per condition. When substrate was incubated for 30 min without the NADPH‐generating system, all values were BLQ or near the lower limits of quantitation. BLQ, below limits of quantitation; FFA, fenfluramine; NADPH, nicotinamide adenine dinucleotide phosphate (hydrogen); nFFA, norfenfluramine

FFA substrate loss and nFFA formation were characterized over time in human liver microsomes (1 mg protein/mL) at substrate concentrations of 1−100 µM (Figure 6). FFA substrate loss was ≤7.7% at all concentrations and times tested (percentage substrate remaining: ≥92.7%). In contrast to observations in S9 liver fractions in the presence of NADPH, there was no detectable substrate loss (~100% substrate remaining) in the absence of NADPH in human liver microsomes (Figure 6A). nFFA formation also required NADPH and increased with incubation time and FFA substrate concentration (Figure 6B). nFFA substrate loss was characterized over time in human liver microsomes (1 mg protein/mL) at substrate concentrations of 0.1−10 µM (Figure 6C). Substrate disappearance ranged from 0−32.7%, with no substrate loss detected in the absence of microsomal protein and substrate loss of 1.2% in the absence of the NADPH‐generating system (controls without microsomal protein: data not shown in Figure 6).

**FIGURE 7:**
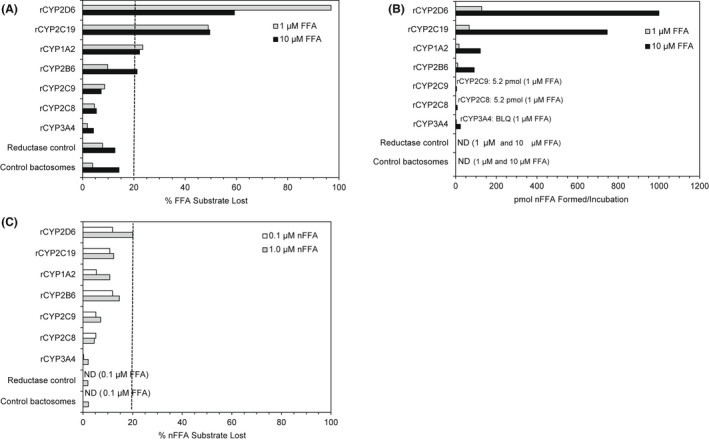
Fenfluramine (FFA) and norfenfluramine (nFFA) substrate loss and nFFA formation by rCYPs. (A) FFA substrate loss (%), (B) nFFA metabolite formation (pmol/incubation), and (C) nFFA substrate loss (%) were determined in recombinant human CYP enzymes expressed in *E*. *coli*. Enzymes were incubated with 1 or 10 µM FFA (A, B) and 0.1 or 1 µM nFFA (C). Controls: membranes from *E. coli*. transfected with empty‐expression plasmid (control bactosome) or with plasmid expressing human NADPH‐cytochrome P450 oxidoreductase but no human CYP enzyme (reductase control). rCYP2B6, rCYP2C8, rCYP2C9, rCYP2C19, and rCYP3A4 were co‐incubated with cytochrome b_5_ reductase. Hashed line indicates 20% threshold. Values are the mean of duplicate determination. BLQ, below the limits of quantification (0.01 μM, which is equivalent to 2 pmol per incubation); FFA, fenfluramine; NADPH, nicotinamide adenine dinucleotide phosphate (hydrogen); nFFA, norfenfluramine; ND, not detected; rCYP, recombinant cytochrome P450

Disappearance of FFA (%) and formation of the metabolite product nFFA (pmol) at 1 μM were observed with rCYP2D6 (97%, 128 pmol), rCYP2C19 (49%, 67 pmol), and rCYP1A2 (23%, 16 pmol); other rCYP450s resulted in <10% FFA loss and <10 pmol nFFA formation (Figure 7A, B). At 10 μM, FFA loss and nFFA formation were observed with rCYP2D6 (59%, 1000 pmol), rCYP2C19 (50%, 745 pmol), rCYP1A2 (22%, 121 pmol), and rCYP2B6 (21%, 91 pmol); in addition, nFFA formation after rCYP3A4 was 24 pmol. Other rCYP450s resulted in <15% substrate loss and <10 pmol nFFA formation at 10 μM. Incubating FFA with individual recombinant enzymes can reveal enzymes capable of metabolizing FFA, but results do not necessarily represent the contribution of each enzyme in a physiological system. Therefore, CYP450‐specific inhibitor studies were conducted in human liver microsomes (Table 2). FFA metabolism was partially inhibited by the selective inhibitors quinidine (CYP2D6, 48%), phencyclidine (CYP2B6, 42%), and furafylline (CYP1A2, 32%). Quinidine (CYP2D6) reduced the formation of nFFA at 55%, 37%, and 51% inhibition at FFA concentrations of 0.1, 1, and 10 µM, respectively (Table 2). While the 42% inhibition by phencyclidine could be due in part to partial inhibition of CYP2D6 by phencyclidine (data not shown), the inhibition by phencyclidine (42%) was nearly as much as the strong CYP2D6 inhibitor quinidine, indicating at least partial metabolism by CYP2B6. Inhibition of FFA metabolism by CYP enzymes supporting less than 10% of the process in vitro was considered insignificant for the identification of enzymes capable of mediating the metabolism of the drug in vivo.

**TABLE 2:** Reduction of FFA demethylation CYP450 enzymes in human liver microsomes

CYP450 enzyme	Selective inhibitor condition		Reduction in FFA demethylation^a^
	Inhibitor	Concentration (µM)	
CYP2D6^b^	Quinidine	0.10, 1, 10	55, 37, 51 (avg 48)
CYP2B6	Phencyclidine	30	42
CYP1A2	Furafylline	10	32
CYP2C9	Tienilic acid	20	14
CYP2C19	Esomeprazole	10	8
CYP3A4/5	Troleandomycin	50	29^c^
	Ketoconazole	0.1, 0.5, 1	None
CYP2A6	Letrozole	10	None
CYP2C8	Gemfibrozil	100	None
CYP2E1	Diethyldithiocarbamate	10	None

Abbreviation: FFA, fenfluramine.

^a^ Percent inhibition by a selective inhibitor (average conditions presented in the case of several test conditions).

^b^ Each row is a separate experiment. Percentages do not sum to 100% due to biological variability and experimental error.

^c^ CYP3A4/5 inhibition by troleandomycin may not be conclusive, as in a subsequent experiment, recombinant human CYP3A4 did not metabolize FFA.

### 3.4 nFFA reaction phenotyping

nFFA (0.1 μM and 1 μM) was incubated with a panel of rCYP450s (CYP1A2, CYP2B6, CYP2C8, CYP2C9, CYP2C19, CYP2D6, and CYP3A4). rCYP1A2, rCYP2B6, rCYP2C19, and rCYP2D6 each metabolized nFFA by 10%−20%, with smaller contributions from the other enzymes (Figure 7C). The low level of esomeprazole inhibition of nFFA formation by rCYP2C19 (8%) indicated a limited contribution of the enzyme to FFA metabolism to nFFA (and clearance; Table 2). Taken together, the recombinant enzyme and specific chemical inhibition data point to CYP2D6, CYP2B6, and CYP1A2 as major contributors to FFA metabolism to nFFA (and clearance), with a possibility of minor contributions from the other enzymes.

### 3.5 Transporter substrate determination

FFA and nFFA were not substrates of the BCRP, OAT1, OAT3, OCT2, MATE1, MATE2‐K, or P‐gp transporters (Table 3). In bi‐directional permeability assays, net efflux ratios for BCRP‐expressing MDCKII cells were below the established 2‐fold threshold for substrate potential at all concentrations of FFA or nFFA (Table 3). Similarly, in a transcellular uptake assay in HEK293 cells expressing OAT1, OAT3, OCT2, MATE1, or MATE2K, and in Caco‐2 cells (for P‐gp), uptake or efflux ratios for all concentrations of both FFA and nFFA were below the threshold value of 2 (Table 3).

**TABLE 3:** Transcellular transporter assays to determine substrate potential of FFA or nFFA for drug transporters

Cell Lines Transfected With Drug Transporter	Probe (concentration, µM)	FFA		nFFA	
		Concentrations (µM)	Maximum Ratio^a^	Concentrations (µM)	Maximum Ratio^a^
MDCKII‐BCRP	Prazosin (1 µM)	0.1, 1, 10	1.51	0.1, 1, 10	1.42
HEK293‐OAT1	[^3^H]‐p‐aminohippurate (1 μM)	0.1, 1, 10	1.22	0.1, 1, 10	1.13
HEK293‐OAT3	[^3^H]‐estrone−3‐sulfate (50 nM)	0.1, 1, 10	1.15	0.1, 1, 10	1.74
HEK293‐OCT2	[^14^C]‐metformin (10 μM)	0.1, 1, 10	1.09	0.1, 1, 10	1.16
HEK293‐MATE1		0.1, 1, 10	1.10	0.1, 1, 10	1.00
HEK293‐MATE2K		0.1, 1, 10	1.40	0.1, 1, 10	1.50
Caco−2—P‐gp		1, 10, 100	0.943	1, 10, 100	1.02

Abbreviations: BCRP, breast cancer resistance protein; FFA, fenfluramine; HEK, human embryonic kidney cells; MATE, multidrug and toxin extrusion; MDCKII, Madin‐Darby canine kidney cells; nFFA, norfenfluramine; OAT, organic anion transporter; OCT, organic cation transporter.

^a^ Ratio of probe net flux or uptake in transporter‐expressing cells to empty‐vector control cells; for P‐gp assays, efflux ratio across Caco‐2 cells was measured in the absence and presence of the P‐gp inhibitor valspodar (10 µM). A threshold ≥2‐fold suggests that an investigational drug is a transporter substrate.

## 4 DISCUSSION

Determining the contribution of CYP450 enzymes to the biotransformation and elimination of FFA and nFFA is an important component of characterizing potential DDIs for FFA in the context of the multi‐ASM regimens used to treat DS.^34^ This study is the first to characterize the victim potential of FFA by in vitro metabolite identification, CYP450 reaction phenotyping, and drug transporter substrate determination according to recent FDA guidance for industry.^24^ Our results suggest that FFA was metabolized to nFFA by CYP2D6, CYP2B6, and CYP1A2, with potential contributions by CYP2C9, CYP2C19, and CYP3A4/5.

In a companion study to the current analysis, neither FFA nor nFFA significantly inhibited or induced CYP450 enzymes, suggesting minimal perpetrator potential at intended clinical doses (0.2−0.7 mg/kg/day, maximum 26 mg/day) (Prescribing information: https://www.fintepla.com/). The absolute oral bioavailability of FFA is reported to be 68%−83%,^35^, ^36^ with extensive metabolism by N‐dealkylation to nFFA,^7^ as confirmed by the current study. Most orally administered, radiolabeled doses of FFA are recovered in the urine as FFA, nFFA, and other metabolites (e.g., glucuronide conjugates of the diol metabolite).^12^ All metabolites recovered in urine are also present in plasma, with marked species differences noted in earlier reports.^12^, ^37^ For example, deamination was not a major metabolic route in the rat, whereas both N‐dealkylation and deamination were important metabolic pathways in the dog, and deamination was observed in the mouse.^12^ Little radioactivity was reported to be excreted in feces in these studies. Taken together with the current study, these results suggest that FFA has multiple mechanisms of elimination. Thus, FFA metabolism is unlikely to be critically affected by inhibition of a single pathway of metabolism.

In a recent clinical DDI study, FFA was co‐administered with a DS ASM regimen of stiripentol, clobazam, and valproate in healthy subjects.^38^ This combination inhibited all of the potential metabolizing enzymes described above and had a significant effect on the pharmacokinetics of FFA, increasing C_max_, AUC_0‐t_, and AUC_0‐∞_, while the C_max_ and AUC_0‐t_ of nFFA were reduced. This study suggested a dose adjustment for FFA as a victim drug but suggested there was little propensity for FFA to act as a perpetrator, even in this combination of ASMs with overlapping metabolic pathways.^38^ Stiripentol is approved for the treatment of Dravet syndrome in the US and Europe. Stiripentol in combination with clobazam and/or valproate inhibits several CYPs (most notably, strong inhibition of CYP2C19 and CYP3A4, with additional inhibition of CYP1A2, CYP2C9, CYP2D6, and CYP2B6; clobazam also inhibits CYP2D6 and valproate weakly inhibits CYP2C9), suggesting the potential for DDIs with other ASMs.^39^, ^40^, ^41^ FFA is partially metabolized by CYP1A2, CYP2B6, and CYP2D6, with the possibility of some additional metabolism by CYP2C9, CYP2C19, and CYP3A4. Thus, the inhibition of multiple CYPs by the stiripentol regimens could result in inhibition of FFA metabolism and an increase in plasma FFA concentrations. Given that all 6 of the FFA‐metabolizing CYPs are inhibited by stiripentol, adding FFA to stiripentol‐containing regimens likely represents a worst‐case scenario potential for DDIs. A downward adjustment of FFA dose is required when prescribed with stiripentol plus clobazam (Prescribing information: https://www.fintepla.com/).

In metabolic stability experiments, FFA showed some NADPH‐independent metabolism in human liver and intestinal S9 fractions, but formation of nFFA and all metabolites formed in liver microsomes from all species required NADPH and/or UDPGA (both components of the cofactor mix). There was no evidence of chemical hydrolysis or non‐enzymatic reactions. Liver microsomes contain CYP450s and uridine diphosphate glucuronosyltransferases (UGTs) that mediate phase 1 and 2 biotransformation reactions requiring NADPH or UDPGA, respectively, whereas S9 fractions contain both microsomal and cytosolic components, including NADPH‐independent mediators of phase 1 and 2 biotransformation reactions.^42^ Notably, formation of nFFA and all six metabolites identified in rat, dog, and human S9 fractions required NADPH, including glucuronidated metabolites C1, C3, and C6 observed only in rat and/or dog. Previous in vivo studies have identified glucuronides of FFA in plasma and urine of human volunteers, as well as mouse, dog, and rat.^12^ The decline in absence of NADPH and the structure of the ketone metabolite would certainly be consistent with metabolism by monoamine oxidase (MAO). MAO was not addressed because in vivo data indicate that most of the clearance of FFA is driven by CYP450 or renal excretion. The loss of substrate in the absence of cofactors along with the structure of the ketone raises the possibility of metabolism by MAO. However, in addition to the increase in FFA reported in the single‐dose DDI study with stiripentol, clobazam, and valproate, at steady state in the patient population, the coadministration of 0.1 mg/kg FFA twice daily with stiripentol plus clobazam, with or without valproate, is expected to result in a 166% increase in FFA AUC_0‐24_ (Prescribing information: https://www.fintepla.com/). The 166% increase is approximately 2.7‐fold which means stiripentol/clobazam took away 69% (100% [1/2.7]) of clearance. In addition, renal clearance accounts for 3% to 10% of the dose excreted as FFA (Prescribing information: https://www.fintepla.com/). As stiripentol/clobazam do not inhibit MAO or renal clearance, approximately 80% of the clearance is accounted for in vivo; the maximum possible amount remaining for metabolism by MAO would be 20% and unlikely to be of clinical significance. Taken together, these results suggest that nFFA and C1−C6 require CYP450s for formation, with the glucuronidated metabolites requiring sequential phase 1 and phase 2 reactions. The appearance of NADPH‐independent substrate loss in rat S9 fractions at the 10‐µM but not the 1‐µM dose suggests that doses used are resulting in CYP450‐independent metabolism in rat and other species evaluated, as was observed in another study.^16^

**FIGURE 8:**
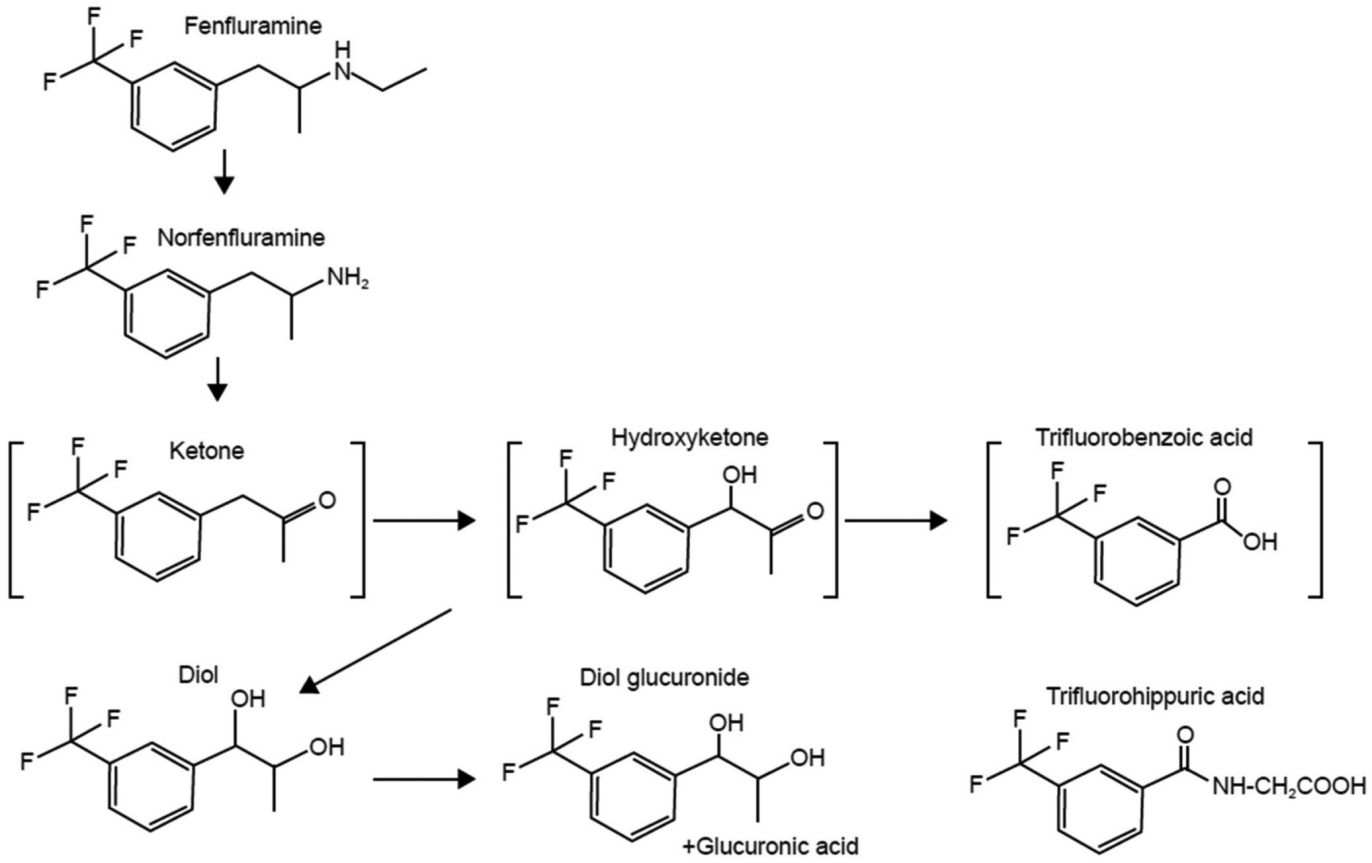
Metabolic pathway of fenfluramine. Adapted from Brownsill 1991^9^

A proposed metabolic pathway for FFA is shown in Figure 8, adapted from Brownsill 1991.^9^ In accordance with prior reports, in our study, only FFA, nFFA, and the N‐oxygenation product of nFFA were found in human liver S9 fractions. All metabolism of FFA appears to lead to nFFA as the first step. None of the observed metabolites could be formed without removal of the N‐ethyl group to form nFFA. Evidence of dehydrogenation, hydroxylation, and glucuronidation was observed in rat and dog but not human S9 fractions. These findings are consistent with prior reports showing that the primary pathway of FFA metabolism is N‐dealkylation to nFFA.^9^, ^12^ Similar to our observations, species differences have been noted in the catalytic activity of enzymes in the CYP1A, CYP2C, CYP2D, and CYP3A families.^43^ These species differences are concordant with in vivo studies performed by Marchant and colleagues. Dealkylation of FFA to nFFA was reported in all species, but deamination of both nFFA and FFA was observed to be a major metabolic pathway in humans. Deaminated products in urine and plasma resulted in the formation of inactive polar metabolites for urinary excretion.^12^ Lack of deaminated metabolites in our in vitro study suggests that the deamination observed in vivo takes place after phase 1 and phase 2 reactions in liver and intestine, probably independently of CYP450s. Rat and dog did not metabolize FFA in the same way as humans. FFA appears to be more extensively and rapidly metabolized in some species relative to humans.^12^, ^44^ Enzymes from all of these families, especially CYP2D6, were observed to contribute to the metabolism of FFA in our studies. Our reaction phenotyping approach provides data to support and extend prior investigations of FFA metabolism by CYP450s, which used indirect methods and isolated investigations of specific CYP450s. Prior reports supported a general role for CYP450s in FFA metabolism,^45^ identified cases where adding FFA to existing regimens resulted in DDIs,^46^ and supported a role for CYP2D6 and CYP1A2 in dexfenfluramine and/or l‐fenfluramine metabolism.^17^, ^47^, ^48^ Reports were contradictory or unclear on CYP2C, CYP3A4, and to our knowledge, CYP2B6 was not specifically investigated in prior reports. Thus, using reaction phenotyping and studies recommended in the US Food and Drug Administration’s most recent Guidance for Industry,^24^ our data extend the prior reports by suggesting a role for CYP2B6 in FFA metabolism, with potential contributions by CYP2C9, CYP2C19, and CYP3A4/5. To our knowledge, this study is the most comprehensive, systematic study published to date on the victim potential of FFA and nFFA because of its adherence to the Guidance to Industry published by FDA and EMA.

FFA was not a likely substrate of any of the drug transporters investigated (BCRP, OAT1, OAT3, OCT2, MATE1, MATE2, P‐gp). Overexpression of transporters in the brain and excretory organs has led to some subtherapeutic ASM plasma levels, especially in refractory epilepsies.^49^ Lamotrigine, an ASM sometimes prescribed in LGS and/or DS, is a P‐gp and a BCRP substrate, but the ASMs phenytoin, phenobarbital, carbamazepine, valproate, topiramate, and levetiracetam did not show substrate capacity for P‐gp and BCRP.^50^ For FFA and nFFA, net efflux or uptake ratios calculated in the current study were outside the threshold range for substantial DDI potential (net efflux or uptake ratio <2). This observation, coupled with hepatic first‐pass metabolism, suggests that the victim potential of FFA is unlikely to arise from drug transporter interactions.

This study is limited by its in vitro design. In addition, pharmacogenetic variants in CYP450s and drug transporters could affect metabolism, although data indicate that polymorphisms of individual CYP450s are unlikely to significantly influence FFA pharmacokinetics (Zogenix, data on file). The high concentration of phencyclidine used as an inhibitor of CYP2B6 may also have the potential to inhibit CYP2D6. Finally, the reason for the non‐NADPH‐dependent loss of substrate (observed most notably in the human liver preparations compared with other species) remains to be established, especially since the MS data indicate NADPH dependency. The most likely explanations include residual NADPH and UDPGA in the fresh S9 preparation and the high signal‐to‐noise ratio afforded by MS compared to the metabolic stability experiments.

It should be noted that some drug metabolism studies incorporate relative activity factor analyses to account for system variations and relative enzyme abundance when using a recombinant CYP approach. For the FFA reaction phenotyping we followed current FDA recommendations that two methods are to be used: enzyme inhibition in HLM/hepatocytes and the recombinant CYPs. We agree that application of the relative activity factor improves extrapolation of in vitro results to in vivo. The results of the two methods we report converge on three major CYPs with indication of possible minor contributions from additional enzymes. The three major enzymes comprise inducible and non‐inducible CYPs, as well as polymorphically and non‐polymorphically expressed CYPs. We consider this information sufficient for addressing a concern of potential susceptibility of FFA to DDI due to a single or a limited number of clearance pathways. Were the results different, application of the relative activity factor would have been considered.

The apparent half‐lives of the parent drug loss were significantly different between 1 and 10 µM and were variable in the species tested, suggesting potentially different apparent Km values among species. To derive the intrinsic clearance from apparent half‐life obtained at a single concentration, it is important to ensure that the concentration is well‐below the apparent Km; alternatively, both 1 and 10 µM should be considered. In human liver S9 incubations, at 10 μM FFA with cofactors, the percent of the drug remaining was 24.4%, 8.8%, and 9.9% at 120, 60, and 30 min, respectively. At 1 μM FFA, the percent of the drug remaining was 20.9%, 11.1%, and 1.9% at 120, 60, and 30 min, respectively. In the absence of the cofactors the percent of the drug remaining was 22.9% and 21.6% in incubations of 10 and 1 μM FFA, respectively. These data are fairly close for the two drug concentrations examined. Aside from that observation, we proceeded on the side of caution and used the data for the lower concentration of the drug, 1 μM, for the evaluation of the half‐life of FFA. Our data support differences in FFA half‐life values obtained in the rat and dog were larger than in the human at two concentrations of the drug.

In the metabolic stability and metabolite identification experiments, UDPGA was included as the cofactor for UGT, but pore forming agents (e.g., alamethicin) were not included in the incubations. The effect of alamethicin on the activity of UGT enzymes is attributed to alamethicin‐mediated formation of pores in the microsomal membrane and an increased access of the UGT substrate to the microsomal lumen where the enzyme binding site is exposed. The addition of alamethicin could increase enzymatic activity of the UGT enzymes. In our studies, FFA was found not to be a substrate for the uptake transporters recommended in the current FDA guidance; therefore, providing an additional route of access of the drug to the enzyme could overestimate its UGT‐mediated clearance.

In conclusion, the in vitro studies described in the current report suggest that FFA may have victim potential, which can manifest when the drug is co‐administered with other ASM regimens containing drugs with substantial effects on combinations of CYP450 family enzymes. The CYP450s with victim potential are CYP2D6, CYP2B6, CYP1A2, and (to a lesser degree) CYP2C9, CYP2C19, and CYPA4/5.

## DISCLOSURE

P.M. and B.B. are employees of, and own stock in, Zogenix, Inc., S.S. is a consultant for Zogenix, Inc., B.W.O., P.B.L., S.M., and M.C. are consultants for Zogenix, Inc., and are employees of Sekisui XenoTech.

## AUTHOR CONTRIBUTION

Participated in research design: BWO, Conducted experiments: SM, Contributed new reagents or analytical tools: N/A, Performed data analysis: SM, Wrote or contributed to the writing of the manuscript: PM, SS, BB, MC, PBL, SM, BWO.

## Data Availability

Zogenix is in the process of establishing a data sharing policy. Written requests for data by legitimate investigators/researchers/clinicians may be submitted to Zogenix, Inc. These requests will be considered on a case‐by‐case basis and reviewed for appropriateness.

## References

[1] Andrade R , Barnes NM , Baxter G , et al. Hydroxytryptamine receptors (version 2019.4) in the IUPHAR/BPS guide to pharmacology database. IUPHAR/BPS Guide Pharmacol CITE. 2019;2019(4):1–31.

[2] Devinsky O , King L , Schwartz D , Conway E , Price D . Effect of fenfluramine on convulsive seizures in CDKL5 deficiency disorder. Epilepsia. 2021;62:e98‐e102. 33979451 10.1111/epi.16923 PMC8360137

[3] Devinsky O , Verducci C , Thiele EA , et al. Open‐label use of highly purified CBD (Epidiolex®) in patients with CDKL5 deficiency disorder and Aicardi, Dup15q, and Doose syndromes. Epilepsy Behav. 2018;86:131‐137. 30006259 10.1016/j.yebeh.2018.05.013

[4] Lagae L , Schoonjans AS , Gammaitoni AR , Galer BS , Ceulemans B . A pilot, open‐label study of the effectiveness and tolerability of low‐dose ZX008 (fenfluramine HCl) in Lennox‐Gastaut syndrome. Epilepsia. 2018;59:1881‐1888. 30146701 10.1111/epi.14540

[5] Lagae L , Sullivan J , Knupp K , et al. Fenfluramine hydrochloride for the treatment of seizures in Dravet syndrome: a randomised, double‐blind, placebo‐controlled trial. Lancet. 2019;394:2243‐2254. 31862249 10.1016/S0140-6736(19)32500-0

[6] Zaccara G , Giovannelli F , Giorgi FS , Franco V , Gasparini S , Tacconi FM . Do antiepileptic drugs increase the risk of infectious diseases? A meta‐analysis of placebo‐controlled studies. Br J Clin Pharmacol. 2017;83:1873‐1879. 28370224 10.1111/bcp.13296 PMC5555858

[7] Beckett AH , Brookes LG . The absorption and urinary excretion in man of fenfluramine and its main metabolite. J Pharm Pharmacol. 1967;19(suppl):42S‐49S. 4383855

[8] Beckett AH , Salmon JA . Pharmacokinetics of absorption, distribution and elimination of fenfluramine and its main metabolite in man. J Pharm Pharmcol. 1972;24:108‐114. 10.1111/j.2042-7158.1972.tb08942.x 4401963

[9] Brownsill R , Wallace D , Taylor A , Campbell B . Study of human urinary metabolism of fenfluramine using gas chromatography‐mass spectrometry. J Chromatogr. 1991;562:267‐277. 2026697 10.1016/0378-4347(91)80584-y

[10] Campbell DB . Gas chromatographic measurement of levels of fenfluramine and norfenfluramine in human plasma, red cells and urine following therapeutic doses. J Chromatogr. 1970;49:442‐447. 5430408 10.1016/s0021-9673(00)93657-4

[11] Campbell DB , Turner P . Plasma concentrations of fenfluramine and its metabolite, norfenfluramine, after single and repeated oral administration. Br J Pharmacol. 1971;43:465p‐466p. PMC1665871 5158239

[12] Marchant NC , Breen MA , Wallace D , et al. Comparative biodisposition and metabolism of ^14^C‐(+/‐)‐fenfluramine in mouse, rat, dog and man. Xenobiotica. 1992;22:1251‐1266. 1492418 10.3109/00498259209053154

[13] European Medicines Agency . Guideline on the investigation of drug interactions, 2012.

[14] Food and Drug Administration . In vitro drug interaction studies ‐ cytochrome P450 enzyme‐ and transporter‐mediated drug interactions guidance for industry, 2020.

[15] Zientek MA , Youdim K . Reaction phenotyping: advances in the experimental strategies used to characterize the contribution of drug‐metabolizing enzymes. Drug Metab Dispos. 2015;43:163‐181. 25297949 10.1124/dmd.114.058750

[16] Nassar AE , King I , Paris BL , et al. An in vitro evaluation of the victim and perpetrator potential of the anticancer agent laromustine (VNP40101M), based on reaction phenotyping and inhibition and induction of cytochrome P450 enzymes. Drug Metab Dispos. 2009;37:1922‐1930. 19520774 10.1124/dmd.109.027516

[17] Gross AS , Phillips AC , Rieutord A , Shenfield GM . The influence of the sparteine/debrisoquine genetic polymorphism on the disposition of dexfenfluramine. Br J Clin Pharmacol. 1996;41:311‐317. 8730977 10.1046/j.1365-2125.1996.03178.x PMC2042591

[18] Rowland NE , Carlton J . Neurobiology of an anorectic drug: fenfluramine. Prog Neurogibol. 1986;27:13‐62. 10.1016/0301-0082(86)90011-0 3526413

[19] Martin P , Czerwiński M , Limaye PB , Ogilvie BW , Smith S , Boyd B . In vitro evaluation suggests fenfluramine and norfenfluramine are unlikely to act as perpetrators of drug interactions. Pharmacol Res Perspect. 2022;10 :e00959. doi:10.1002/prp2.959 PMC9124818 35599347

[20] Parkinson A , Mudra DR , Johnson C , Dwyer A , Carroll KM . The effects of gender, age, ethnicity, and liver cirrhosis on cytochrome *P*450 enzyme activity in human liver microsomes and inducibility in cultured human hepatocytes. Toxicol Appl Pharmacol. 2004;199:193‐209. 15364537 10.1016/j.taap.2004.01.010

[21] Pearce RE , McIntyre CJ , Madan A , et al. Effects of freezing, thawing, and storing human liver microsomes on cytochrome P450 activity. Arch Biochem Biophys. 1996;331:145‐169. 8660694 10.1006/abbi.1996.0294

[22] Ogilvie BW , Usuki E , Yerino P , Parkinson A . In vitro approaches for studying the inhibition of drug‐metabolizing enzymes responsible for the metabolism of drugs (reaction phenotyping) with emphasis on cytochrome P450. In: Rodrigues AD , ed. Drug‐Drug Interactions: Drugs and the Pharmaceutical Sciences. 2nd ed. Informa Healthcare USA; 2008:231‐358.

[23] Bjornsson TD , Callaghan JT , Einolf HJ , et al. The conduct of in vitro and in vivo drug‐drug interaction studies: a Pharmaceutical Research and Manufacturers of America (PhRMA) perspective. Drug Metab Dispos. 2003;31:815‐832. 12814957 10.1124/dmd.31.7.815

[24] US Food and Drug Administration . In vitro drug interaction studies — cytochrome P450 enzyme‐ and transporter‐mediated drug interactions. Guidance for industry, 2020.

[25] Feng B , Mills JB , Davidson RE , et al. In vitro P‐glycoprotein assays to predict the in vivo interactions of P‐glycoprotein with drugs in the central nervous system. Drug Metab Dispos. 2008;36:268‐275. 17962372 10.1124/dmd.107.017434

[26] Giacomini KM , Huang SM , Tweedie DJ , et al. Membrane transporters in drug development. Nat Rev Drug Discovery. 2010;9:215‐236. 20190787 10.1038/nrd3028 PMC3326076

[27] Izumi S , Nozaki Y , Komori T , et al. Substrate‐dependent inhibition of organic anion transporting polypeptide 1B1: comparative analysis with prototypical probe substrates estradiol‐17*b*‐glucuronide, estrone‐3‐sulfate, and sulfobromophthalein. Drug Metab Dispos. 2013;41:1859‐1866. 23920221 10.1124/dmd.113.052290

[28] Vermeer LMM , Isringhausen CD , Ogilvie BW , Buckley DB . Evaluation of ketoconazole and its alternative clinical CYP3A4/5 inhibitors as inhibitors of drug transporters: the in vitro effects of ketoconazole, ritonavir, clarithromycin, and itraconazole on 13 clinically‐relevant drug transporters. Drug Metab Dispos. 2016;44:453‐459. 26668209 10.1124/dmd.115.067744

[29] Yamazaki M , Neway WE , Ohe T , et al. In vitro substrate identification studies for p‐glycoprotein‐mediated transport: species difference and predictability of in vivo results. J Pharmacol Exp Ther. 2001;296:723‐735. 11181899

[30] Harding SD , Sharman JL , Faccenda E , et al. The IUPHAR/BPS Guide to PHARMACOLOGY in 2019: updates and expansion to encompass the new guide to IMMUNOPHARMACOLOGY. Nucleic Acids Res. 2018;46:D1091‐1106. doi:10.1093/nar/gkx1121 29149325 PMC5753190

[31] Alexander SPH , Christopoulos A , Davenport AP , et al. THE CONCISE GUIDE TO PHARMACOLOGY 2021/22: G protein‐coupled receptors. Br J Pharmacol. 2021;178:S27‐S156. doi:10.1111/bph.15538 34529832

[32] Alexander SPH , Fabbro D , Kelly E , et al. The concise guide to pharmacology 2021/22: Enzymes. Br J Pharmacol. 2021;178:S313‐S411. doi:10.1111/bph.15542 34529828

[33] Alexander SPH , Kelly E , Mathie A , et al. The concise guide to pharmacology 2021/22: Transporters. Br J Pharmacol. 2021;178:S412‐S513. doi:10.1111/bph.15543 34529826

[34] Wallace A , Wirrell E , Kenney‐Jung DL . Pharmacotherapy for Dravet syndrome. Paediatr Drugs. 2016;18:197‐208. 26966048 10.1007/s40272-016-0171-7

[35] Bever KA , Perry PJ . Dexfenfluramine hydrochloride: an anorexigenic agent. Am J Health Syst Pharm. 1997;54:2059‐2072. 9377205 10.1093/ajhp/54.18.2059

[36] Richards R . The metabolism and kinetics of fenfluramine, its optical isomers and a structural analogue, benfluorex. Doctoral thesis, in: A thesis submitted for the degree of doctor of philosophy with the University of Surrey, Universtiy of Surrey, Surrey, UK. 1985.

[37] Bruce RB , Maynard WR Jr . Fenfluramine metabolism. J Pharm Sci. 1968;57:1173‐1176. 5662056 10.1002/jps.2600570717

[38] Boyd B , Smith S , Gammaitoni A , Galer BS , Farfel GM . A phase I, randomized, open‐label, single‐dose, 3‐period crossover study to evaluate the drug‐drug interaction between ZX008 (fenfluramine HCl oral solution) and a regimen of stiripentol, clobazam, and valproate in healthy subjects. Int J Clin Pharmacol Ther. 2019;57:11‐19. 30336805 10.5414/CP203276 PMC6298132

[39] Giraud C , Treluyer JM , Rey E , et al. In vitro and in vivo inhibitory effect of stiripentol on clobazam metabolism. Drug Metab Dispos. 2006;34:608‐611. 16415114 10.1124/dmd.105.007237

[40] ONFI^®^ (clobazam) tablets, for oral use, CIV [prescribing information] ONFI^®^ (clobazam) oral suspension, CIV [prescribing information]. Lundbeck; 2016.

[41] Wen B , Ma L , Rodrigues AD , Zhu M . Detection of novel reactive metabolites of trazodone: evidence for CYP2D6‐mediated bioactivation of *m*‐chlorophenylpiperazine. Drug Metab Dispos. 2008;36:841‐850. 18238857 10.1124/dmd.107.019471

[42] Parkinson A , Ogilvie BW , Buckley DB , Kazmi F , Czerwinski M , Parkinson O . Biotransformation of xenobiotics. In: Klaassen CD , ed. Casarett & Doull’s toxicology, the basic science of poisons. McGraw‐Hill Companies Inc; 2013:185‐376.

[43] Martignoni M , Groothuis GM , de Kanter R . Species differences between mouse, rat, dog, monkey and human CYP‐mediated drug metabolism, inhibition and induction. Expert Opin Drug Metab Toxicol. 2006;2:875‐894. 17125407 10.1517/17425255.2.6.875

[44] Caccia S , Ballabio M , Guiso G , Rocchetti M , Garattini S . Species differences in the kinetics and metabolism of fenfluramine isomers. Arch Int Pharmacodyn Ther. 1982;258:15‐28. 7138139

[45] Anelli M , Fracasso C , Bergami A , Ferrarese A , Garattini S , Caccia S . Effect of d‐fenfluramine on the indole contents of the rat brain after treatment with different inducers of cytochrome P450 isoenzymes. Psychopharmacology. 1995;118:188‐194. 7617807 10.1007/BF02245839

[46] Fogelson DL . Fenfluramine and the cytochrome P450 system. Am J Psychiatry. 1997;154:436‐437. 10.1176/ajp.154.3.436b 9054800

[47] Haritos VS , Ching MS , Ghabrial H , Ahokas JT . Measurement of dexfenfluramine metabolism in rat liver microsomes by gas chromatography‐mass spectrometry. J Chromatogr B Biomed Sci Appl. 1997;693:327‐336. 9210436 10.1016/s0378-4347(97)00025-x

[48] von Moltke LL , Greenblatt DJ , Ciraulo DA , et al. Appetite suppressant drugs as inhibitors of human cytochromes P450: in vitro inhibition of P450–2D6 by D‐ and L‐fenfluramine, but not phentermine. J Clin Psychopharmacol. 1998;18:338‐341. 9690701 10.1097/00004714-199808000-00015

[49] Lazarowski A , Czornyj L , Lubienieki F , Girardi E , Vazquez S , D’Giano C . ABC transporters during epilepsy and mechanisms underlying multidrug resistance in refractory epilepsy. Epilepsia. 2007;48(suppl 5):140‐149. 17910594 10.1111/j.1528-1167.2007.01302.x

[50] Romermann K , Helmer R , Loscher W . The antiepileptic drug lamotrigine is a substrate of mouse and human breast cancer resistance protein (ABCG2). Neuropharmacology. 2015;93:7‐14. 25645391 10.1016/j.neuropharm.2015.01.015

